# Diagnosing norms and norm change in rural Bangladesh: an exploration of gendered social norms and women’s empowerment

**DOI:** 10.1186/s12889-023-17213-2

**Published:** 2023-11-24

**Authors:** Shivani Chandramohan, Allison P. Salinger, Amanda S. Wendt, Jillian L. Waid, Md. Abul Kalam, Maryann G. Delea, Dawn L. Comeau, Shafinaz Sobhan, Sabine Gabrysch, Sheela Sinharoy

**Affiliations:** 1 Behavioral, Social, and Health Education Sciences, Rollins School of Public Health, Emory University, 1518 Clifton Rd NE, Atlanta, GA 30322 USA; 2 Hubert Department of Global Health, Rollins School of Public Health, Emory University, Mailstop 1518-002-7BB Clifton Rd NE, Atlanta, GA 30322 USA; 3 Research Department 2, Potsdam Institute for Climate Impact Research (PIK), Member of the Leibniz Association, P.O. Box 60 12 03, 14412 Potsdam, Germany; 4 Heidelberg Institute of Global Health, Heidelberg University, Im Neuenheimer Feld 324, 69120 Heidelberg, Germany; 5 Bangladesh Country Office, Helen Keller International, Rd No 82, Dhaka, 1212 Bangladesh; 6 Gangarosa Department of Environmental Health, Rollins School of Public Health, Emory University, 1518 Clifton Rd NE, Atlanta, USA; 7 Charité – Universitätsmedizin Berlin, corporate member of Freie Universität Berlin and Humboldt-Universität Zu Berlin, Institute of Public Health, Charitéplatz 1, 10117 Berlin, Germany

**Keywords:** Norms diagnosis, Social norms, Empowerment, Norm change, Norm abandonment, Agency

## Abstract

**Background:**

Gender-transformative public health programs often aim to address power inequities between men and women and promote women’s empowerment. However, to achieve transformative change, it is necessary to first identify the underlying norms that perpetuate these power imbalances. The objective of our study was to use Bicchieri’s theory of social norms and model of norm change to identify gendered norms and evidence of norm change amongst participants of the Food and Agricultural Approaches to Reducing Malnutrition (FAARM) trial in rural Sylhet Division, Bangladesh.

**Methods:**

We conducted ten life history interviews, 16 key informant interviews, and four focus group discussions with women and men in communities within the FAARM study site in rural, north-eastern Bangladesh. We performed a thematic analysis as well as a relational analysis of the data.

**Results:**

We found that social norms dictated the extent and ways in which women participated in household decisions, the locations they could visit, and their autonomy to use household resources. We also found evidence of changes to gendered social norms over time and the desire amongst some men and women to abandon restrictive norms. Certain intersecting factors, such as education and employment, were identified as facilitators and barriers to women’s empowerment and the related gendered expectations.

**Conclusions:**

Our findings corroborate existing norms literature, which highlights the strong role social norms play in influencing women’s empowerment and behaviour. Our study provides an example of rigorous qualitative methodology that others may follow to assess gendered social norms that can be targeted for transformative change.

**Supplementary Information:**

The online version contains supplementary material available at 10.1186/s12889-023-17213-2.

## Background

Women’s empowerment has long been recognized for its intrinsic value and, in addition, for its important contributing role in health outcomes, particularly for women and children [1–3]. Empowerment is defined as a process of change allowing individuals to gain control over their own lives, enabling them to make strategic life choices previously denied to them [3, 4]. Evidence suggests that empowerment increases women’s access to and use of health services, their likelihood of receiving effective health care, and participation in decision making for health-seeking behaviours [1, 2, 5]. This positively impacts the whole household as women are traditionally the gatekeepers of family health [6–9]. In contrast, the disempowerment of women not only affects their own households, but also impacts health on a societal level [2, 9–11]. When women are unable to participate in leadership positions or contribute to national decisions and policymaking, both women and men receive less effective healthcare [2, 12] and suffer a range of other adverse health outcomes [13–15].

Agency, a crucial component of the empowerment process, is the ability to pursue goals, take action, express voice, and influence and make decisions [3, 16]. Whilst decision making is the most commonly measured aspect of agency, other aspects of agency include financial autonomy, freedom of movement, and collective action [4]. Expressions of agency facilitate change at an individual level through motivation and sense of purpose (for example, preference or desire to participate in a given decision-making event) [3]. A woman’s inability to express agency and autonomy is often due to restrictive norms, which can drive behaviours that hinder access to opportunities, resources and power [7, 17].

### Diagnosis of norms & norm change

Whilst several frameworks exist for social norms analysis (SNA) [18], each with their own strengths and limitations, we selected Bicchieri’s theory of norm change for our purposes as it specifically focuses on the process and stages of norm change and attempts to identify what motivates collective patterns of behaviour [19]. Bicchieri defines a social norm as ‘a rule of behaviour such that individuals prefer to conform to it on the conditions that they believe; 1) a majority of people in their reference network conform to it (empirical expectation) and 2) most people in their reference network believe they ought to conform to it (normative expectation)’ [19].What separates a social norm from a moral rule, habit or custom is its interdependent and conditional nature, meaning other people’s actions and opinions matter to one’s choice. Hence, the social expectations (empirical and normative expectations) individuals have about the beliefs and behaviours of their reference network, or the range of people they care about when making decisions, are the driving factors of social norms [19].

One key indicator of social norms is the existence of sanctions [19]. Sanctions are social enforcements of behaviour that reiterate societal expectations and can be negative or positive [19, 20]. Social norms are sustained by sanctions, as the threat of negative sanctions allow social norms to dictate behaviour [21]. According to Bicchieri, sanctions are crucial to diagnosing social norms as they highlight the existence of socially conditional expectations, which distinguish social norms from personal normative beliefs (that is, an individual’s beliefs about what ought to be done regardless of the behaviours or expectations of others). Personal normative beliefs may happen to be similar amongst individual members of a network but are not contingent upon the actions or perceived beliefs of others in the network and, therefore, do not constitute social norms [19]. The consequences of norm transgressions, through the deployment of sanctions, indicate strong normative expectations. If a behaviour elicits a form of social condemnation or praise, through the deployment of sanctions, this indicates the presence of a social norm.

**Fig. 1:**
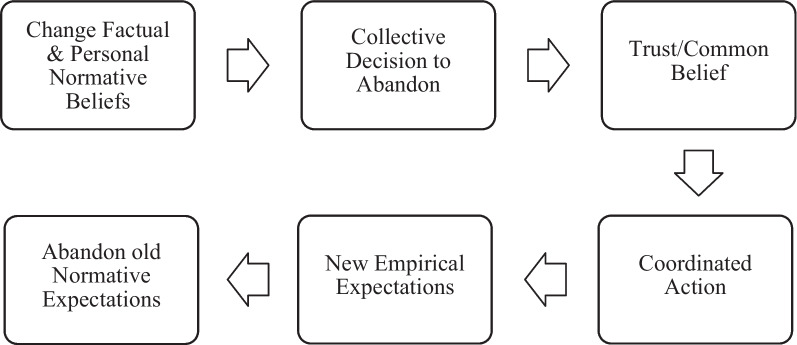
Bicchieri’s framework of the steps of norm change [19]

A change in norms is essentially a change in social expectations [19]. Social norms are deeply rooted within communities, and the emergence of new gendered social norms is a complex process requiring changes in social expectations and approval amongst members of the reference network, which, in the case of gendered social norms, typically includes both men and women. Norm change can involve the creation of new norms or the abandonment of existing norms. As shown in Fig. 1, Bicchieri details norm abandonment as a six-step process consisting of: 1) Change in factual and personal normative beliefs, where factual beliefs are those that individuals understand to be true and personal normative beliefs relate to ‘what should be done’; 2) Collective decision to abandon, 3) Trust/common belief that others will also change the practice or behaviour, 4) Coordinated action to change a practice or behaviour, 5) Creation of empirical expectations (i.e., beliefs about how others in the reference network behave), and 6) Abandonment of old normative expectations (i.e., beliefs about how others in the reference network believe one ought to behave) [19]. To abandon maladaptive norms, members of the reference network must recognize the problems created by existing norms and have shared reasons to change.

### Social norms and gender

Social norms, or informal behavioural rules, govern collective patterns of behaviours within communities through social expectations, compliance, and sanctions [16, 19, 22]. In patriarchal societies, social norms, particularly those dictating gender roles, can perpetuate systems of inequality by limiting the rights and powers of women [16, 23–25]. This pattern ultimately leads to the continued disempowerment of women [16]. At the same time, studies show empowered women gain greater control of their expressions of agency, such as making their own decisions and choices, leading to changes in social norms [16, 26]. It is essential to understand existing social norms and the mechanisms by which norms change, to understand and facilitate the process of empowerment [3, 27].

Several studies have examined components of existing social norms in relation to behaviours, practices, and gender roles in low-and middle-income countries (LMICs). For example, a study assessing intimate partner violence (IPV) in Nepal found community-based expectations and perceptions were better predictors of women’s risk of IPV when compared to individual-level measures of women’s attitudes [28]. In the case of female genital mutilation/cutting (FGM/C) in Senegal and The Gambia, the results of a qualitative study found the practice was continuing due to social pressure from elders, rather than religious customs or perceived health benefits [29]. A study assessing exclusive breastfeeding in Mali found that mothers’ perceptions of the community’s behaviour greatly predicted her own, indicating a strong reliance on social expectations [30]. Similarly, a qualitative study identifying established norms in rural West Africa found the statement ‘everyone agrees’ was the predominant reason why individuals engaged in normative behaviours aligned with traditional gender roles [18]. Whilst these studies demonstrate the utility of assessing social norms related to gender, few studies have assessed existing social norms and explored the changes in norms over time, particularly in the context of LMICs [18]. Previous studies have focused on changing specific behaviours through behaviour change interventions, or quantifying behaviour change, rather than considering and exploring changes in the factors that facilitate changes in behavioural outcomes [18, 31].

### Study context and aims

Despite progress, Bangladesh continues to face high levels of malnutrition as well as issues of gender inequality, which are linked and can be tied to disempowerment [3, 32]. Homestead food production (HFP) programs use a group-based approach, often with women's groups, to provide training and support for home gardening, poultry rearing, and improved nutrition and hygiene practices in order to improve women’s and children’s nutrition outcomes [33]. To evaluate the impact of a HFP program on malnutrition, a cluster-randomized control trial was set up in Bangladesh, named Food and Agricultural Approaches to Reducing Malnutrition (FAARM), with linear growth amongst children born to enrolled women in participating communities as its primary outcome [34]. The international non-governmental organisation Helen Keller International implemented this HFP program jointly with the local non-governmental organisation Voluntary Association for Rural Development from 2015 to 2018 in parts of rural Habiganj, Sylhet, Bangladesh. Further details on the FAARM trial have been reported in the study protocol [34].

Women’s empowerment was a key outcome in the FAARM theory of change [34]. Specifically, the FAARM intervention included components that were designed to promote women’s decision-making, participation and leadership in producer groups, and income generation through the sale of surplus produce [34]. Therefore, this qualitative study aimed to examine the prevailing norms that informed behaviours and practices related to women's agency amongst FAARM trial participants. In line with the FAARM theory of change, we were particularly focused on understanding norms informing three key empowerment-related practices: household decision-making, freedom of movement (which is required for group participation), and financial independence. We further aimed to identify whether individual characteristics (e.g., marital status, education, employment) influenced the degree to which women were expected to abide by the prevailing norms for a given gender- or empowerment-related practice. We also explored whether these norms changed over time within FAARM intervention communities.

## Methods

### Study design and participant sampling

This study was a secondary analysis of qualitative data collected in February 2018 and February 2019 as part of the Gender, Agriculture, and Assets Project, Phase Two (GAAP2), which was led by the International Food Policy Research Institute (IFPRI). GAAP2 worked through 13 projects in Asia and Africa, including FAARM, to develop improved indicators for women's empowerment in agriculture. The study design for the qualitative research largely followed protocols that were developed by IFPRI for implementation across the GAAP2 portfolio. Within FAARM, eligibility criteria for women to enrol in the overall trial included being married, access to at least 40 square metres of land, and a self-reported age of 30 years or younger at enumeration in 2014 [34]. Qualitative participants were purposively sampled from both Muslim and Hindu communities, as it was hypothesized that norms and conceptualizations of empowerment may differ by religion.

The first round of qualitative data collection for this study (February 2018) involved focus group discussions (FGDs) and life history interviews. For the first round, two intervention communities were selected based on proximity to the project office and to capture one community that was predominantly Muslim and one that was predominantly Hindu. FGD participants were recruited from a list of enrolled FAARM participants and their husbands, with the help of local staff. Participants invited for life history interviews were married couples (interviewed separately) who had previously participated in the quantitative GAAP2 empowerment survey. During data collection, one husband was not available to participate in the life history interview. Therefore, another couple from a neighbouring community who fit the inclusion criteria was selected instead.

The second round of data collection (February 2019) was carried out to iteratively explore in greater depth issues that had been identified in the data from the first round. Similar methods were used to recruit life history interview participants from two additional intervention communities (one Hindu and one Muslim). In addition, the FGD guide used in the prior round of data collection was adapted to be used in key informant interviews (KIIs). Key informants were project participants who had been selected as group leaders by project staff and their communities. Key informants were able to reflect the local practices of their community and FAARM group.

### Data collection procedures

The life history interviews and KIIs were semi-structured and took place inside participants’ homes. The life history interviews explored general perceptions of empowerment, as well as family structure, norms, and participants’ overall life events as they relate to gender. KIIs included questions about the participant’s role as a leader of a FAARM group, as well as about perceptions of empowerment, with a focus on decision making and freedom of movement. FGDs assessed community perceptions of the ability to express different dimensions of agency as well as norms, gender roles, and women’s empowerment. Each life history interview, KII, and FGD was conducted in Bengali by two trained research staff, one facilitator and one note taker, who were the same gender as the participants. Whilst many participants spoke Sylheti, a local dialect of Bengali, they also spoke mainstream Bengali, and the research team was familiar with key terms and phrases in Sylheti that were relevant to the research topics. Each interview and FGD lasted one to two hours and was audio recorded.

The life history interview and FGD guides were adapted from guides developed by IFPRI to assess empowerment in the context of agricultural interventions. Specifically, the guides were adapted to the local context through an iterative review process between researchers and local program staff prior to data collection. The KII guide (used in the second round of data collection) was based on the previously adapted FGD guide but adjusted for the new format and tailored to the targeted respondents (i.e., group leaders). Example questions include: ‘In households where men and women make most decisions together, how are women in those households perceived by other women and men in their community?’, and ‘Why are certain decisions over production made together or alone?’. Pilot testing was undertaken at the beginning of each round, with adjustments made to the guides to increase understanding and facilitate active participation of respondents. Guides were also updated over the course of data collection through daily debriefings and discussion of findings [35].

### Data management and analysis

Audio recordings were transcribed verbatim by members of the data collection team and then translated into English by an independent translator. Following translation, all documents were de-identified.

To conduct this secondary analysis, transcripts were reviewed and analytic memos were written to record first impressions of the data. After reviewing the transcripts, a codebook was developed using both deductive and inductive codes (Appendix, Supplemental Table 1)*.* We used Christina Bicchieri’s theory of social norms and model of norm change [19] to develop deductive codes related to norms. In addition, we used the conceptual model of women and girl’s empowerment, as designed by KIT Royal Tropical Institute and the Bill & Melinda Gates Foundation (BMGF) [16], to develop deductive codes related to empowerment. This conceptual model was selected for its comprehensiveness in covering many aspects of empowerment. Inductive codes were based on concepts that were identified from the data. The codebook was reviewed and refined through a collaborative process with three of the authors (SC, MGD, and SSS). Once all codes were finalised, transcripts were coded by one analyst (SC) in MAXQDA 2020 [36]. Data were analysed through multiple rounds of coding using thematic analysis [37], in which themes were developed using the coded data to identify patterns related to empowerment and norm change. We used an intersectional approach to examine the influence of individual socio-demographic characteristics, or intersecting factors, on expectations related to women’s conformity with prevailing norms [38]. Relational analyses were conducted using MAXMAPS code co-occurrence models [37], to further identify and visualise links between themes and how they relate to, and influence, the process of empowerment.

### Ethics and informed consent

The data analysed in this study were collected in accordance with relevant guidelines and regulations as part of the FAARM trial (ClinicalTrials.gov: NCT02505711). The study protocol was positively reviewed by the ethics committees of the James P Grant School of Public Health at BRAC University in Bangladesh and Heidelberg University in Germany. FAARM is registered with ClinicalTrials.gov (NCT02505711). All study participants gave written informed consent prior to participation in FAARM, and additionally provided verbal informed consent for interviews and FGDs.

## Results

In total, ten individual life history interviews (representing five married couples), 16 KIIs, and four gender-segregated FGDs were conducted across the two rounds of data collection, with a total of 63 participants (33 women and 30 men). A breakdown of sample characteristics and demographic data can be found in the Appendix (Supplemental Tables 2-5).

The results are organized according to the study’s research aims. First, we present results for the diagnosis of norms related to women’s agency, with a focus on decision making, freedom of movement, and financial independence. We then present individual characteristics that were identified as influencing the degree to which women are expected to abide by prevailing norms. Finally, we review the process of norm change within FAARM intervention communities.

Table 1, below, summarises social norms and expectations present in the study population, and their respective sanctions.

**Table 1:** Summary of the evidence of social norms among study population

**Social norm**	**Empirical expectations**	**Normative expectations**	**Sanctions**^a^
Decision-making	- Women usually take all decisions about household work. […] The women of the locality will never be able to take their own decisions without the consent of their guardians (Male, FGD)	- Women can’t take any major decisions. Many are able to take minor decisions (Male, FGD)	- I: If a woman from a family like that makes such a decision, what would they say about her? Would they say bad things about her? P: They would say bad things about her. She has to ask for permission from her in-laws. She can do that if they agree with her (Female, FGD)
Freedom of Movement	- Women usually take permission before going anywhere after their marriage (Male, FGD)	- Women do not have any work outside, that’s why they should stay in the household (Male, LH) - A woman should take permission and take someone with her (Male, FGD) - Our sisters couldn’t go where we could go. Why would they go outside? They should remain covered (Male, LH) - Women should work like women do, but they cannot go outside of the house [to work] (Male, KII)	- If they [women] go to the marketplace people would comment that the woman is bad. Everyone tries to rectify the women’s behavior. The women who go to the marketplace are ignored by all (Males, FGD) - Those women who go to marketplace or go somewhere alone, the other women say about them that those women are really not good. They do not respect their husbands, parents, fathers-in-law and mothers-in-law, they do not veil and lead a vulgar life. (P5,P8) The other women of the locality think that women who go to the marketplace do not respect their elder brothers, elder sisters or brothers-in-law (Males, FGD) - People will condemn that the wife has gone alone there (Female, LH)
Financial Independence	- Women work in the home (Female, FGD) - I take decisions about the earnings outside the home. How I earn, how I spend – these things. However, women help to earn inside the home. My wife takes the decision about the earnings inside the home (Male, FGD)	- Women’s work should be done by women (Female, FGD) - How earnings should be spent is decided by me usually. She too tells me it is my responsibility to run the family. I should decide how the money would be spent. I give her what is needed (Male, LH)	- I: Suppose I am earning and don’t ask my husband. I spend that independently. […] What will happen if I don’t ask anyone? P: This will not be good. There will [be] no peace in [the] family. (P3) Elders are neither respected nor consulted; following own desire is not good. People will comment on this. (P4) I: If such thing happens, how are those women treated? P: […] We shall not think of her as good (P6) (Females, FGD)

^a^*I* Interviewer, *P* Participant

### Decision making

Social norms dictated the degree to which women were able to participate in household level decisions. Specifically, decision-making processes related to childcare and healthcare were viewed as areas women were allowed to participate in and even lead, whereas decisions around household purchases and agricultural production were seen as outside of the women’s realm. The majority of participants reported that most decisions were often made jointly with their spouses; however, men were ultimately given the final say. Men and women reported limitations on women’s abilities to make their own decisions in overall decision making. One man in an FGD explained, ‘*On this matter, everyone says that women in our area can’t take decisions alone. You can say there is no example of it*’ (Man, FGD). This statement represents an existing empirical expectation related to women’s ability to engage in decision making, as it indicates beliefs about the practices of others in the reference network.

There was evidence of social norms related to women’s behavior around decision making. For example, when discussing independence in decision making, a participant stated, ‘*the newly married women should not do that. They have to listen to their husbands and in-laws in that period of life*’ (Woman, KII). (In-laws are referenced here due to the custom of patrilocality after marriage.) In addition the data provided evidence of sanctions, further strengthening the diagnosis of the social norms influencing women’s decision making as social norms. Sanctions, in the name of honour, drove gendered normative behaviours and served as an impediment to change. When asked what happens to a woman who breaks norms and gender roles, including related to decision-making, both men and women stated they would criticize them, but would not pursue or implement formal sanctions such as in the form of a community hearing. However, respondents also mentioned they will maintain a distant relationship with such ‘bad women’ and avoid interacting with them. One participant mentioned, ‘*The people of the society would spread bad comments about those women. They will say that this woman is bad. Whoever socializes with her will become bad as well*’ (Woman, FGD).

### Freedom of movement

Freedom of movement refers to a woman’s ability to choose where and when she visits locations outside her home. The prevalent norms related to freedom of movement in the study area suggested women were generally restricted from leaving the house unless they obtained permission, were escorted by a male family member, went in a group for religious gatherings, or had an important reason to leave the household. Women reported that their freedom of movement was restricted as they were expected to complete household tasks, whereas men were responsible for tasks outside of the home. Women also reported they were required to have accompaniment when making trips to the market, the doctors’ office to seek non-urgent medical treatment, and their parents’ homes*.* As a result, although decisions relating to food and health were considered to be in a woman’s domain, often it was men who implemented the actions associated with these decisions as women often did not have the freedom to move to locations such as markets, pharmacies, and clinics. A female participant stated, ‘*No. It is not permitted [for women] to go just in front of the house, let alone the marketplace’* (Woman, Life History (LH)).

The data also included evidence of prevalent empirical and normative expectations related to women’s freedom of movement, detailing how people in the community expected women to behave, which revealed that women’s movements were influenced by social norms. For example, according to one male participant, ‘*even now, 80 per cent of women don’t go to market*’ (Man, FGD). This empirical expectation that the majority of women in the community remained at home was shared among most participants, both men and women. Regarding normative expectations, many women and men made statements such as ‘*We [men] can go many places, many houses but they [women] can’t. Because of their honour they can’t live like men*’ (Man, LH), demonstrating that participants also believed that women *should* or were supposed to remain at home.

In terms of women’s freedom of movement, safety was rarely mentioned as a concern. Sanctions, in the form of what others will think and say, were the main barrier to women’s freedom of movement. When questioned on the consequences of women travelling alone, a male participant mentioned, ‘*If women go to the places where they should not go, the people of the society would say that the women are bad and their ancestors were also bad. … But if they go with their guardians, then people would think that they are good women*’ (Man, FGD).

A family’s honour amongst society appeared to depend largely on the actions of the women of the family. Participants reported that deviating from established norms can compromise women’s futures, and, for example, put their marriage at stake, which as we will demonstrate in the next section, would have further implications for women’s access to resources. These informal sanctions often took place in the form of mocking or gossip, for instance according to a female participant, ‘*as a boy, someone can go anywhere as no one would say anything bad about it. But if a girl does that, people would say bad things about it. The society would say bad things as well*’ (Woman, LH). Sanctions, in the form of damage to one’s honour and virtue, were discussed across most of the interviews, even though this topic was never explicitly asked about in the interview guides. For example, one participant explained:

*If anyone goes out without taking permission from their parents, it’s a matter of honour. If my daughter goes outside and anyone teases her, it becomes a matter of shame for me. If I see that going to that place can become good for her, then I will give her permission to roam around, but if I see that it can’t be good at that time, I can’t permit it*. (Woman, FGD)

### Financial independence

In terms of financial independence, or a women’s ability to have control over economic resources, study participants indicated that women often did not have control over the household’s finances as men were the primary income earners. Some women reported engaging in income-generating activities such as sewing or selling eggs from poultry. Whilst they were able to spend money on minor purchases for their children, they needed to consult their husbands on all other purchases. Empirical expectations as they relate to financial independence were that women needed their husband’s permission to spend money, for example, multiple women in an FGD agreed, ‘*They do not allow us even to spend two takas [Bangladeshi currency]’; ‘What we need, they bring. Can’t spend even one taka*.’; *‘We can keep it but cannot spend it.*’ (Women, FGD). Male participants mentioned similar circumstances, ‘*Wife can’t alone decide to spend husband’s income.’; ‘Wife cannot spend husband’s income without permission. She has to inform the reason, like buying vegetables or anything else*’ (Men, FGD). These data suggest that existing normative expectations restrict women’s financial independence, indicating that women’s financial behaviours are influenced by social norms.

The expectation to conform to norms around control over finances can greatly limit women’s ability to be independent. Women must maintain ‘good’ behaviour for fear of negative social sanctions. For instance, one woman explained that women who work were looked down upon by male family members, as men believed it indicated that they (the men) were incapable of providing for their families. Men also mentioned they would feel bad if their sisters or wives had to work outside the home, according to a male participant, ‘*The people would have said why the sisters would work outside despite having four brothers’, ‘It is a matter of honour and respect. The men would make fun of them*’ (Man, LH). There were also negative terms associated with independent women, for example, ‘*I have already said paa lomba mohila can spend their money themselves*’ (Man, FGD). *Paa lomba mohila*, literally long-legged women, is a phrase used in the study area to refer, negatively, to women who go outside of the household frequently and spend money on their own.

### Intersecting factors

Several factors played a role in influencing the degree to which individuals were expected to follow certain social norms. Certain women, depending on marital status, education, employment, location and socio-economic status, were either exempt from or subject to attenuated empirical and normative expectations. These intersecting factors aided in influencing social norms and served as either facilitators of or barriers to women’s empowerment, depending on the context. In other words, these specific intersectionalities modified the extent to which women’s agency-related behaviours were conditional on empirical and normative expectations. At the same time, other intersecting factors, such as religion or age, were not found to be important for influencing expectations (i.e., the same social expectations applied to women across age groups and regardless of religion).

#### Marital status

Participants reported that the behaviour of a wife was seen as a reflection of her husband, and thus many wives were bound to their husband’s rules and standards. For instance, a female participant stated, ‘*It will be bad if I travel around this and that (locality). Now I am a wife. If I do that then people will say, ‘See, the wife of …has come to chitchat’. For that I have to care about my dignity’* (Woman, LH)*.* Some women did not see these practices as being restrictive, and instead accepted these behaviours as a part of the role of a wife, as an FGD participant explained, ‘*Women can go anywhere if their husbands take them’* (Woman, FGD).

Newlywed women were particularly restricted, according to a participant, *‘You have to take permission before going anywhere. Say, I need to go to that house, first I have to take permission from my mother-in-law. It happens when you are married’* (Woman, LH). Whilst young unmarried women were subject to their own restrictions, respondents recalled having more freedom prior to marriage when living in their parents' homes, than as a newlywed under control of their mother-in-law.

In line with this, women who were not wives, such as unmarried or widowed women, appeared to be subject to less stringent restrictions, as the same norms did not apply to them. For example, one participant explained, ‘*The woman who does not have a husband makes her own decision’* (Man, FGD). Similarly, women whose husbands worked abroad also had more decision-making authority, freedom of movement, and financial independence as they had no choice but to take care of their households and do all the tasks themselves. Given that male migration is common in the study region, higher levels of empowerment amongst women whose husbands were migrants seemed to be widely accepted within their communities.

#### Education & employment

In the case of education and employment, both were seen as crucial factors facilitating women’s empowerment, and this was a common theme throughout the interviews. It was often suggested that women could do everything a man does if they were educated or employed. Women who received a higher education or engaged in an income-generating activity had different sets of social expectations compared to less educated and unemployed women, as it was understood that these activities contributed positively to the household. For example, education played a role in allowing a woman to make her own decisions, as a participant noted: *‘The literate [women] can make decisions alone, I mean, the [women] who have education, they can make decisions alone*’ (Man, KII). Similarly, employment and education were important contributors to women’s freedom of movement, and this was recognized by both men and women. Women mentioned they often could not step outside of the house if they did not have a purpose, but if they were going to school, college, or a job, they would be allowed to go outside without express permission or an escort, ‘*Those [women] who have jobs can travel alone*’ (Woman, FGD). Education was also seen as key to granting a woman long-term independence, *‘Education is the reason for the increase of their [women’s] freedom. Women’s tendency to achieve success has increased because of education*’ (Man, FGD).

Employment and education also impacted a woman’s control over finances. For example, one participant stated, *‘A girl can run a family if she has good education and employment’* (Woman, FGD). A participant mentioned that in cases where women earned a supplemental income, they were able to use their personal income on items for themselves or their children, providing them with a source of autonomy: ‘*My wife raises roosters. Sometimes there is some spending that is needed for the children. My wife spends that from her earnings*’ (Man, LH). Women also expressed that they wouldn’t be limited to household chores and could live life on their own terms if employed or educated.

#### Location

Residing in a rural location was considered a barrier to women’s empowerment, as a more restrictive code of conduct is expected in rural areas. Participants perceived that woman living in urban or peri-urban areas had different expectations and were allowed to move freely and make their own independent choices with less judgement. When asked about the origin of the existing expectations around women’s movement, a female participant responded, ‘[…] *it has been running since the age of our ancestors. This code is based on their social norms. This exists only in villages. But you can roam anywhere in towns’* (Woman, FGD). Women mentioned they could not have any aspirations, or even go shopping, as they were from the village and those desires were limited to urban women: *‘They go for shopping. This is the scenario of the cities…The village women hardly go to the marketplaces. They go when they absolutely have to go’* (Woman, KII).

#### Socio-economic status

Similarly, women who were from very rich and very poor families had specific social expectations that were different from those for middle-class women. Expectations were less stringent for the rich due to either their education or urban location. According to participants those facing extreme poverty could leave their homes to work as they had to earn for their families: ‘*Moreover, people will not accept it if women go outside. But the poor women can go outside because they do not have any other option to earn income*’ (Woman, FGD).

### Norm Change

Life history participants were asked to reflect on changes they witnessed from the time they were children, compared to the present. The analysis indicated that the process of change, though slow, was underway amongst the study population. Specifically, the course of norm change was in line with the process of norm abandonment described by Bicchieri [19]. Whilst not each specific step of norm abandonment for each dimension of agency was found amongst the study population, there was evidence of change in regard to some aspects of women’s empowerment.

Step one in Bicchieri’s theory of norm change (change in factual and personal normative beliefs) was apparent in the data, such as when a participant said, ‘*we have a future if we have a job*’ (Woman, FGD). Personal normative beliefs are an individual’s views on what is ‘good’ and what one should do. Most women articulated changes in their personal normative beliefs about the value of education, which as explained above, was viewed positively and played a role in determining the degree to which women had to adhere to social norms informing decision making, freedom of movement, and financial independence behaviors. They mentioned that they regretted not receiving an education and that they were unaware previously of the benefits of education. Women participants stated they did not have good role models, they were not pushed by their families, and hence did not focus on their education when they were children but aimed to change this for their children. Similarly, the overall trend of increased education amongst women appears to have facilitated changes in beliefs regarding women of the new generation and their abilities to make their own choices, which aligns with findings from our intersectionality analyses. A participant mentioned, ‘*We thought about our honour before. Nowadays an educated girl knows about her rights, she can come on strong about not marrying a particular person if she already has chosen another man. We could not do that back then*’ (Woman, LH).

Step two (collective decision to abandon) involves a desire amongst the reference network, as a whole, to change a given collective behaviour. Data from our study participants showed no clear evidence of a collective decision to abandon traditional gender roles. However, evidence suggested an agreement to allow for exceptions to the norms regarding women’s employment, which may have important implications for women’s financial independence or women’s empowerment generally. Participants appeared to value instances where both a husband and wife served as income-earning members to bring in additional income for the family, and better support their children. For example, male participants stated, ‘*In today’s age, it is difficult to sustain without both husband and wife working. But many changes occurred compared to the past. … This change has been going on for 20 years.’; ‘There were no garment factories in the past. But now, both husband and wife are working with having kids. They work at garment factories in Dhaka [Capital of Bangladesh]*’ (Men, FGD).

The attainment of step three (trust and common belief that abandoning a norm is, indeed, beneficial) was evidenced by statements such as, ‘*People are being educated slowly. And now, some say that women are running the country, their views have to be considered*’ and ‘*women and men have equal rights*’ (Women, FGD). These quotes indicate that people were starting to recognize women’s rights. Participants reported that they believed these changes were good for their families and the country. One participant also stated, ‘*If the women go out, they can do anything*’ (Woman, FGD).

Step four (coordinated action or steps taken towards the common goal of norm abandonment) was being achieved with the help of government programs as well as initiatives by non-governmental organisations (NGOs). According to respondents, ‘*Bangladesh government has given them [women] equal rights now*’ (Man, LH).

‘*But now the government of Bangladesh is ensuring gender equality. Now there is no problem because of the environment. Getting a job was very hard for a woman before. But the number of jobs increased over time and now women can live their lives with their children by doing these jobs. They can now earn their living by doing jobs. … Now there are garment factories and different private companies where they can get jobs. They can now earn their living in some way*’ (Man, KII).

However, there was a lack of evidence detailing coordinated action for change within the communities themselves. At the time of the study, change was driven by perilous situations such as poverty; however, there was no evidence of a proactive push for changing gendered social expectations for women. When questioned on why certain women can work, a participant suggested, ‘*Because of poverty. Women are forced to go out to work because of deficiency in the family’* (Man, KII).

Evidence also suggested that step five, or new empirical expectations, had at least started to occur, as new empirical expectations were already at play and involved men and women witnessing more examples of empowered women. This signals a shifting of perceptions amongst participants regarding how women behave. One participant reported, ‘*In our area, in Habiganj, many women go to different places like the circus and political meetings. Currently women are playing important roles in our area, I mean, they are not staying behind now*’ (Man, LH).

Table 2 details participant responses relating to changing empirical expectations on dimensions of agency, such as decision making, mobility, and financial independence.

**Table 2:** Examples of changing empirical expectations

Social norm	Evidence of changing empirical expectation
Decision-Making	“Because everyone understands that it is better to have discussions together. People are realizing this slowly. People are realizing now that women are also human.” (Females, FGD) “No family ever progresses without woman. Some families are developed entirely by women. […] A family is built upon woman.” (Females, FGD) “It’s changing slowly. In the past, men didn’t connect with women in the family. Presently, it is changing, even it is a little.” (Female, FGD)
Freedom of Movement	“Women’s movement has increased compared to previous times.” (Male, FGD) “[Women] are now going to the marketplaces. But it was not the same before. As it is ‘Digital Bangladesh’, the women are free here. They go to marketplaces to buy culinary produce. That’s the reason.” (Male, KII) “Why more women are going out now? Because the government is run by a woman now. We follow our government.” (Female, KII) “The literacy rate of the country is increased, people have jobs, they have to go in different places. Mobility has changed nowadays. Environment has also changed.” (Male, KII)
Financial Independence	“Presently, a husband will be glad if his wife gets employed.” (Male, FGD) “Now, women have more power. […] Now, Bangladesh has digitized, government has taken many steps. Women’s literacy has increased, job opportunity has increased for women in different sectors.” (Male, KII)

Overall, participants mentioned there were more empowered women at the time of the study compared to when they were children: ‘*People in the past didn’t allow women to do anything they want. Now women can do anything. Women now understand what would be good and what would be bad for them. They help each other to change their situation*’ (Woman, KII). However, participants recognized that only a few women within their reference network fit the description or perception of an empowered woman: ‘*Everybody says, on this matter, that 10 per cent women of our area are now working or employed … Q: Are they going outside more? A: Hasn’t it increased? Now they go to the cities for their jobs*’ (Man, FGD).

There was also evidence of changed empirical expectations, as a result of government programs and policies. Many participants described witnessing initiatives from the government and NGOs, such as TV programs and training opportunities that give women more visibility and demonstrate that women should have more of a voice and broader roles in society, aided in creating new empirical expectations surrounding women’s empowerment. Similarly, the presence of women in leadership positions in Bangladesh’s political sphere, such as having a female prime minister, created positive perceptions of empowered women.

Evidence from this study suggests that the final step of norm abandonment, the abandonment of old normative expectations, was still in progress in study communities. An empowered woman was defined by most participants as, ‘*educational qualification is high, they have jobs, they earn money—these are empowered women*’ (Man, KII). Most respondents, both men and women, had positive perceptions about empowered women, who could make and implement their own choices, and believed an increase in empowered women was beneficial for society. They stated, ‘*I want them to get jobs. They would be able to make their own future if they have jobs. I want women to receive an education. Their economic condition will be improved in all terms*’ (Man, KII). Whilst participants agreed that women should be empowered, employed, and educated, there was no drive for women to actively change all the normative expectations surrounding traditional gender roles. Hence, whilst some normative expectations have changed, such as those suggesting women can have jobs outside of the home, other normative expectations remain unchanged, such as those declaring they must dress modestly and work in ‘good’ jobs. ‘*There is nothing to think bad about the men whose wives have jobs. Women from reputed families have NGO jobs.’; ‘There is no problem if women have jobs. But they have to be modest and in veil [head covering] at their work. No one thinks negatively about the men whose wives have jobs*’ (Men, FGD).

#### Future aspirations

Most of the men and women interviewed indicated a strong desire for future change. They stated that without their current elder generation (such as their parents), they would be the future elders, and hence could allow and advocate for changes to occur. They wanted their daughters to be educated, employed, and empowered as they believed ‘*women take the society forward*’ (Man, FGD). They recognized opportunities for women were changing and understood the need for change – but were still bound by norms due to the persistence of their community elders. In particular, one male respondent mentioned, ‘*I want their [women’s] betterment, I hope their condition will be better in future. At that time elders will not exist. Our generation will replace them [the elders]. There will be none to criticize them. There will be no one to criticize them if they return home from work at dusk. Women will work freely then*’ (Man, KII).

## Discussion

This study contributes to our understanding of women's experiences in rural Bangladesh relating to social norms and empowerment. Findings suggest there were prevalent social norms dictating aspects of decision making, freedom of movement, and financial independence. Evidence from this study indicates both men and women were not ready for women to abandon norms as a whole, but some aspects of each norm were already shifting. Most of this change was driven by the desire to improve participants’ current economic conditions, and resulted in changes in some norms; however, these changes may not be indicative of increases in women’s empowerment across all of rural Sylhet. It is also important to note that whilst most participants aspired for future change and believed women were capable of being empowered, these statements were not necessarily reflective of the beliefs they had of women within their own households. Whilst participants had positive perceptions of women’s empowerment overall, they did not always believe that empowerment was attainable for themselves or their wives due to social expectations.

We identified certain intersecting factors, such as education, employment, marital status, socio-economic status, and geographic location (urban vs. rural) that could influence the degree to which women were expected to abide by gendered social norms and social expectations. For instance, women could make decisions about their finances if their husbands were employed abroad, leave the house if they were going to work or school, and earn an income to support their family under dire situations. These factors served as both facilitators and barriers to women’s empowerment and related practices. Existing research in Bangladesh indicates similar findings, noting that expectations or restrictions placed on women were often relaxed for educational purposes – increasing their mobility, and decision-making autonomy [39–42]. However, outside of educational circumstances, norms were still enforced [40, 42]. There were no notable differences found by religion or age of the respondent, though the latter may be due to the relatively young age of women in our study population.

There have been advances in altering gendered social norms and there was a desire, amongst some women and men, to abandon restrictive norms. Most study participants had a positive perception of empowerment and empowered women but believed empowerment was unattainable by the current generation, and instead were hopeful this change could occur for their daughters or granddaughters. Participants also recognized education and employment as a woman’s path to empowerment, but they struggled to alter existing community beliefs and seek opportunities. However, overall, the divide between women and men seemed to have reduced over time and participants reported that more people were starting to view men and women as equals.

Our findings are consistent with other studies, particularly game-theoretic portrayals of the social dynamics of norms abandonment, which suggest that ‘in interdependent, larger groups, the choice of each member depends on the choice of all members’ [43]. From a game-theoretic perspective, until a ‘tipping point’ is reached – when a sufficiently large proportion of the community is ready to abandon the harmful practice (for example, restrictive gender norms, gender inequality) – the harmful practice will persist [44]. The initial stages of change that are signalled through our findings from this research suggest that whilst segments of the population have abandoned some harmful gendered beliefs, and general attitudes are changing, coordinated collective abandonment has not been achieved. Findings from other norms abandonment examinations of harmful practices such as FGM/C, child marriage, and foot binding suggest that norm abandonment is possible, but only through the coordination of collective abandonment within a given community [43, 45].

Despite advancements, there continued to be strongly held beliefs surrounding a woman’s honour, particularly that a woman’s engagement in non-normative behaviours can bring dishonour on her family. Similarly, many participants distinguished between behaviours or social expectations for ‘good’ and ‘bad’ women. These findings are in accordance with the existing norms literature indicating that although individuals may have different personal beliefs, social norms continue to be stronger predictors in terms of governing behaviours [20, 28, 29].

It is a limitation of this study that the data collection tools were not designed for SNA and were thus not specifically tailored to the study aims; rather, we leveraged existing data to examine prevailing norms amongst the study population. Additionally, social desirability bias may have influenced participants’ responses. Finally, an inclusion criterion for the FAARM trial was being married and a self-reported age of 30 years or younger at enumeration, hence our study does not capture the perspectives of older or unmarried women. The study’s strengths include the use of qualitative methods to provide an emic perspective on norm change over time, as well as the use of data from both men and women, as empowerment studies often do not include men. This article also fills existing gaps by contributing to the body of literature on social norms.

Using Bicchieri’s norms diagnostic framework and norms abandonment model to guide this analysis is both a strength and limitation of the study. Key strengths include leveraging of theory to diagnose gender-related norms and examine evidence of norm abandonment, as well as using established theoretical concepts to inform the development of an analytical codebook and framework. However, Bicchieri’s norms abandonment model has not been tested in this context, which reflects a limitation of this study. To our knowledge, norm abandonment models have not been used in studies of social norms in relation to empowerment.

Our results indicate that some steps in the norms abandonment model were not precisely reflective of people’s experiences with social norms change in our site. There were often overlaps between steps of Bicchieri’s model and what was indicated in our results; the model depicts the process of norms abandonment as being linear and step-wise, but our findings rather indicated an iterative norms abandonment process. Consequently, this study did not provide empirical evidence to support the specific model of norm abandonment, as outlined by Bicchieri. Our results suggest that norms abandonment is a more complex, non-linear, and potentially cyclical process than that depicted in Bicchieri’s norms abandonment model: a critique that has been voiced by other authors as well [46]. However, the external validity of our results is uncertain, and the iterative process suggested by our findings may differ from the process of norms abandonment and norm change in other contexts. Recent critique of SNA has called for critical assessment of the use of this approach—developed in high income countries—in LMICs for development practice [46]. This paper constitutes one example of an application of SNA in a development context and offers critical suggestions for adaptations of the theoretical model.

Women’s empowerment in the context of LMICs is limited by restrictive social norms and aspects of gender inequality resistant to change. Identifying existing social norms, their linkages to local context and culture, and how they continue to change, is crucial to designing appropriate interventions to empower women, bring about gender equality, and improve health outcomes [47]. Addressing the false beliefs that can sustain harmful collective practices (for example, correcting bias in perceptions regarding empirical and normative expectations), and coordinating collective abandonment within a given community are also important considerations for intervention design [27, 43, 48, 49]. Given that sensitivity to social expectations may be greater amongst those who lack agency or autonomy [27, 49], it is also important to incorporate intervention techniques that are specifically designed to increase the agency required to abandon harmful social norms and coordinate collective abandonment within a given community. Future studies and interventions aiming for women’s empowerment should combine awareness of the importance of norm change with opportunities for income-generating activities and education in order to effectively overcome restrictions on women imposed by gendered social norms [48, 50]. It is essential to improve agency and address these perceptions and social expectations to create lasting change.

### Supplementary Information

**Additional file 1:** **Appendix.** **Supplemental Table 1.** Analytic Codebook. **Supplemental Table 2.** Round 1 Focus Group Discussion (FGD) Participant Characteristics by sex and religion. **Supplemental Table 3.** Round 1 Life History (LH) Participant Characteristics. **Supplemental Table 4.** Round 2 Life History (LH) Participant Characteristics. **Supplemental Table 5.** Round 2 Key Informant Interview (KII) Participant Characteristics.

## Data Availability

The datasets used and/or analysed during the current study are available from the corresponding author on reasonable request.
