# Supplementary material for: Diagnosing norms and norm change in rural Bangladesh: an exploration of gendered social norms and women’s empowerment
Source: BMC Public Health. 2023 Nov 24;23:2337. doi: 10.1186/s12889-023-17213-2 (PMC10675851; doi:10.1186/s12889-023-17213-2)
Supplement: Supplementary file 1 — Additional file 1: Appendix. Supplemental Table 1. Analytic Codebook. Supplemental Table 2. Round 1 Focus Group Discussion (FGD) Participant Characteristics by sex and religion. Supplemental Table 3. Round 1 Life History (LH) Participant Characteristics. Supplemental Table 4. Round 2 Life History (LH) Participant Characteristics. Supplemental Table 5. Round 2 Key Informant Interview (KII) Participant Characteristics. [file 12889_2023_17213_MOESM1_ESM.docx]

# Appendix

**Supplemental Table 1.** Analytic Codebook

| **Level 1 Code** | **Level 2 Code** | **Level 3 Code** | **Definition** | | |
| --- | --- | --- | --- | --- | --- |
| Empowerment | Decision-Making | Finances | Women influencing and making decisions about finances | | |
| Empowerment | Decision-Making | Children & Household Needs | Women influencing and making household decisions | | |
| Empowerment | Freedom of Movement | Accessible Locations | Women's autonomy to move freely to access needs and facilities | | |
| Empowerment | Resources | Social Capital | Women's relations and social networks that provide tangible and intangible value and support | | |
| Empowerment | Resources | Knowledge & Skills | Women's knowledge and skills related to income and labor and their abilities to apply those knowledge and skills | | |
| Empowerment | Resources | Education | Women's education levels and their impacts on empowerment | | |
| Empowerment | Resources | Financial Independence | Women's control over economic resources | | |
| Empowerment | Critical Consciousness |  | Women's application of critical thinking skills to examine their current situation and develop a deeper understanding of their reality. | | |
| Empowerment | Safety & Security |  | Women's freedom from acts or threats of violence (physical or sexual), coercion, harassment, or force | | |
| Empowerment | Leadership |  | Women assume leadership positions, effectively participate, and support women's leadership in informal and formal initiatives and organizations | | |
| Empowerment | Influence | Social | When a person's emotions, opinions, or behaviors are affected by others (social hierarchy, support, pressure) | | |
| Empowerment | Honor / Respect |  | Factors influencing women's perceptions of their respect and honor | | |
| Norms | Empirical | Self | Individuals say they are going to do (what I am going to do) | | |
| Norms | Empirical | Others (Empirical Expectations) | Individuals believe large subset of group/ population conforms to the norm (what others do) | | |
| Norms | Empirical | 2nd Order | Individuals mention how others believe they behave (what others believe I/others do) | | |
| Norms | Normative | Self (Personal Normative Belief) | Personal belief about what is right (or wrong) (what I should do) | | |
| Norms | Normative | Others (Normative Belief) | Individuals believe society should behave in a certain way(what others should do) | | |
| Norms | Normative | 2nd Order (Normative Expectations) | Individuals believe large subset of group/ population expects them to conform to the norm (what others believe I/others should do) | | |
| Norms | Factual Belief |  | One's belief/opinion is about the state of the world (could be right or wrong) | | |
| Norms | Descriptive Norm |  | Empirical expectations are sufficient to motivation action/behavior |  |  |
| Norms | Social Norms |  | The mention of both normative and empirical expectations within the same quote (conditional on normative expectations) | | |
| Norms | Sanctions | Positive | Introduce positive sanctions for non-compliance | | |
| Norms | Sanctions | Negative | Introduce negative sanctions for non-compliance | | |
| Norm Change | New decisions | Desire to comply | Change factual and normative decisions (willingness to continue/change behaviors in the future) | | |
| Norm Change | New decisions | Past performance | Change factual and normative decisions (differences in behaviors compared to previous experiences) | | |
| Norm Change | Collective action |  | Collective decision to change | | |
| Norm Change | Collective Action | Self-Efficacy (self) | Self-efficacy to change one's own behavior | | |
| Norm Change | Collective Action | Self-Efficacy (collective) | Self-efficacy to contribute to collective "norm changing" action | | |
| Norm Change | Collective Action | Intrinsic Collective Agency | Mention of locus of control | | |
| Norm Change | New expectations | Normative | Create normative expectations | | |
| Norm Change | New expectations | Empirical | Create empirical expectations | | |
| Intersectionality | Socioeconomic status |  | Reference to socioeconomic status as an intersecting factor | | |
| Intersectionality | Marital status |  | Reference to marital status as an intersecting factor | | |
| Intersectionality | Household composition |  | Reference to household composition as an intersecting factor | | |
| Intersectionality | Location |  | Reference to location (urban vs periurban vs rural) as an intersecting factor | | |
| Intersectionality | Religion |  | Reference to religion as an intersecting factor | | |
| Intersectionality | Age |  | Noted differences between elders, adults, and children around behaviors of interest, age as an intersecting factor | | |
| Institutional Structures | Relations |  | The interactions & relations - including conflicts, support, hostility and communication with key actors that shape women's experiences | | |
| Institutional Structures | Gender Roles/ Dynamics |  | Collectively held beliefs of how women/men should behave& interact inside/ outside the household (esp regarding division of labor, noted differences between men & women) | | |
| Type of Response | Confusion |  | Respondent found the question confusing or did not understand. Enumerator may have repeated the question multiple times or provided additional examples. | | |
| Type of Response | Distress |  | Respondent expressed anger or distress in reaction to a question. | | |
| Type of Response | Irrelevance |  | Respondent said that a particular question was not relevant to her specific situation or that the question was irrelevant to her context as a whole. | | |
| Type of Response | Attitudes |  | A respondent’s personal positions or leanings related to gender/women’s empowerment | | |
| Implementation Factors | Facilitators |  | Factors that drive/enable the practice of desired behaviors | | |
| Implementation Factors | Behaviors |  | Factors that prevent the practice of desired behaviors | | |

**Supplemental Table 2.** Round 1 Focus Group Discussion (FGD) Participant Characteristics by sex and religion

| Gender | Religion | Age | Number of Children | Age of Children (years) |
| --- | --- | --- | --- | --- |
| Female | Hindu | 24 | 2 | 6, 5 |
|  |  | 25 | 0 | - |
|  |  | 25 | 2 | 9, 6 |
|  |  | 26 | 2 | 7, 4 |
|  |  | 26 | 2 | 8, 4 |
|  |  | 24 | 2 | 8, 5 |
|  |  | 22 | 2 | 4, 6 months |
|  |  | 20 | 1 | 1 |
|  | Muslim | 19 | 1 | 2, 0.5 |
|  |  | 20 | 1 | 2, 0.4 |
|  |  | 22 | 2 | 12, 5 |
|  |  | 23 | 2 | 12, 5 |
|  |  | 25 | 2 | 4, 1 |
|  |  | 22 | 4 | 9, 7, 4, 2 |
|  |  | 22 | 1 | 4 |
|  |  | 26 | 2 | 12, 7 |
|  |  | 26 | 2 | 7, 4 |
|  |  | 27 | 4 | 13, 12, 9, 4 |
|  |  | 30 | 3 | 11, 9, 3 |
|  |  | 20 | 2 | 4, 1.5 |
| Male | Hindu | 20 | 2 | 2, 1 month |
|  |  | 30 | 0 | - |
|  |  | 36 | 4 | 12, 10, 8, 6 |
|  |  | 40 | 2 | 7, 1 |
|  |  | 37 | 2 | 7, 4 |
|  |  | 28 | 1 | 3 |
|  |  | 26 | 2 | 8, 5 |
|  |  | 36 | 2 | 2, 1 month |
|  |  | 30 | 2 | 8, 4 |
|  | Muslim | 25 | 2 | 12, 8 |
|  |  | 29 | 2 | 10, 4 |
|  |  | 28 | 4 | 12, 10, 8, 6 |
|  |  | 27 | 2 | 4, 2 |
|  |  | 28 | 2 | 8, 2 |
|  |  | 27 | 1 | 4 |
|  |  | 30 | 2 | 5, 2 |
|  |  | 33 | 3 | 7, 3, 2 |

**Supplemental Table 3.** Round 1 Life History (LH) Participant Characteristics

| ID | Gender | Religion | Occupation | Age | Highest Level of Schooling Attended | Duration in Village |
| --- | --- | --- | --- | --- | --- | --- |
| LH1 | Female | Hindu | N/A | 30 | Class 5 | 10 years |
| LH2 | Female | Hindu | N/A | 31 | Class 4 | 16 years |
| LH3 | Female | Muslim | Housewife, Tailor | 28 | Class 4 | 14 years |
| LH4 | Female | Muslim | N/A | 26 | Class 3 | 6 years |
| LH5 | Female | Muslim | Housewife | 28 | Class 4 | 11 years |
| LH1 | Male | Hindu | Agriculture, Day labor | 40 | Class 1 | 40 years |
| LH2 | Male | Hindu | Agriculture, Day labor | 38 | Class 8 | 38 years |
| LH3 | Male | Muslim | Day Labor, Driver (tom-tom) | 27 | None | 27 years |
| LH4 | Male | Muslim | Agriculture, Business | 41 | None | 36 years |
| LH5 | Male | Muslim | Agriculture, Fish Business | 33 | None | 33 years |

**Supplemental Table 4.** Round 2 Life History (LH) Participant Characteristics

| ID | Gender | Religion | Occupation | Age | Highest Level of Schooling Attended | Duration in Village |
| --- | --- | --- | --- | --- | --- | --- |
| LH1 | Female | Muslim | Housewife | 30 | Class 5 | 15 years |
| LH2 | Female | Muslim | Housewife | 27 | Class 4 | 12 years |
| LH3 | Female | Hindu | Housewife | 27 | None | 15 years |
| LH4 | Female | Muslim | Housewife | 30 | None | 10 years |
| LH1 | Male | Muslim | Day labor / agriculture | 55 | None | 22 years |
| LH2 | Male | Muslim | Day labor / agriculture | 32 | Class 2 | 20 years |
| LH3 | Male | Hindu | Agriculture | 40 | None | 40 years |
| LH4 | Male | Hindu | Job/Salesman | 35 | Class 8 | 25 years |

**Supplemental Table 5.** Round 2 Key Informant Interview (KII) Participant Characteristics

| ID | Gender | Religion | Occupation | Age | Highest Level of Schooling Attended |
| --- | --- | --- | --- | --- | --- |
| KII 1 | Female | Muslim | Housewife | 34 | Class 4 |
| KII 2 | Female | Hindu | Housewife | 33 | Class 5 |
| KII 3 | Female | Hindu | Housewife | 30 | Class 7 |
| KII 4 | Female | Muslim | Housewife | 31 | Class 3 |
| KII 1 | Male | Hindu | Unskilled day labor | 34 | Class 5 |
| KII 2 | Male | Muslim | Skilled day labor | 39 | Class 7 |
| KII 3 | Male | Hindu | Business | 37 | Class 7 |
| KII 4 | Male | Muslim | Farmer | 39 | Class 5 |
